# A protocol for a living mapping review of global research funding for infectious diseases with a pandemic potential – Pandemic PACT

**DOI:** 10.12688/wellcomeopenres.21202.2

**Published:** 2024-10-02

**Authors:** Olena Seminog, Rodrigo Furst, Thomas Mendy, Omid Rohanian, Shanthi Levanita, Zaharat Kadri-Alabi, Nusrat Jabin, Georgina Humphreys, Emilia Antonio, Adrian Bucher, Alice Norton

**Affiliations:** 1 Policy and Practice Research Group, Pandemic Sciences Institute, University of Oxford, Oxford, England, UK; 2 Computational Health Informatics Lab, Department of Engineering Science, University of Oxford, Oxford, England, UK; 3 Green Templeton College, University of Oxford, Oxford, England, UK; 4 UK Collaborative on Development Research, London, UK; 5 GloPID-R Research and Policy Team, Policy and Practice Research Group, Pandemic Sciences Institute, University of Oxford, Oxford, England, UK

**Keywords:** Pandemic preparedness, priority diseases, research funding, coordination, global health policy

## Abstract

The COVID CIRCLE initiative Research Project Tracker by UKCDR and GloPID-R and associated living mapping review (LMR) showed the importance of sharing and analysing data on research at the point of funding to improve coordination during a pandemic. This approach can also help with research preparedness for outbreaks and hence our new programme the Pandemic Preparedness: Analytical Capacity and Funding Tracking Programme (Pandemic PACT) has been established. The LMR described in this protocol builds on the previous UKCDR and GloPID-R COVID-19 Research Project database with addition of the priority diseases from the WHO Blueprint list plus initial additions of pandemic influenza, mpox and plague. We capture data on new funding commitments directly from funders and map these against a core ontology (aligned to existing research roadmaps). We will analyse regularly collated new research funding commitments to provide an open, accessible, near-real-time overview of the funding landscape for a wide range of infectious disease and pandemic preparedness research and assess gaps. The periodicity of updates will be increased in the event of a major outbreak. We anticipate that this LMR and the associated online tool will be a useful resource for funders, policy makers and researchers. In the future, our work will inform a more coordinated approach to research funding by providing evidence and data, including identification of gaps in funding allocation with a particular focus on low- and middle-income countries.

## Introduction

The COVID-19 pandemic exposed significant problems with traditional funding and research structures in a pandemic response context, leading to slow research activation and duplicative efforts. The scale and urgency of the research response was challenging to coordinate.

To guide investments early in the pandemic, global identification of research priorities was provided by the World Health Organization (WHO) in collaboration with GloPID-R through the development of the Coordinated Global Research Roadmap on Novel Coronavirus and the ongoing activities of the WHO R&D Blueprint team ^1^. In vaccine development and clinical trials, substantial innovative and rapid research progress was made. Research funding, however, was poorly coordinated, resulting in fragmentation of the funding landscape and a proliferation of underpowered, heterogeneous studies with little impact in terms of actionable results to improve health outcomes ^2, 3^. The global distribution of research funding and activities was uneven, with the majority of research projects taking place in high-income countries (HICs) during the pandemic ^2^, despite the heavy burden COVID-19 placed on low- and middle- income countries (LMICs).

The need to map research funding to global and regional prioritisation strategies led to the collaborative funding bodies GloPID-R and UKCDR establishing the COVID-19 Research Coordination and Learning Initiative (COVID CIRCLE). COVID CIRCLE was underpinned by a set of principles to align research funders in a coordinated effort to support high-quality research addressing the most pressing global needs in epidemics and pandemics ^4^. One of COVID CIRCLE’s major achievements is the COVID-19 Research Project Tracker ^5^. This live database and associated living mapping review ^2^ of funded research projects on COVID-19 aligned to the key policy roadmaps with over 40,000 online views helping funders and researchers identify gaps and opportunities and inform future research investments and coordination needs. This work informed global funding decisions and its importance highlighted in the R&D Preparedness Ecosystem: Preparedness for Health Emergencies Report to the Global Preparedness Monitoring Board ^6^ and in the WHO Overall Achievements Report ^7^ released for the first year’s research response to the pandemic.

The shortcomings witnessed during the COVID-19 pandemic reflect wider challenges in the health R&D ecosystem (which pre-dated the pandemic) and persist in the post-pandemic period. These have implications for research effectiveness in terms of research conduct and uptake of research outputs; R&D governance; health research capacity; and research financing ^8^. The continued limitations to monitoring health R&D investments, in particular, pose a significant challenge to ensuring accountability and the efficient use of scarce resources for health ^9^. 

Building on the clear ‘use case’ for rapid tracking and analysis of research at the point of funding, in order to enable evidence-informed decision making, we have formed the Pandemic Preparedness: Analytical Capacity and Funding Tracking Programme (Pandemic PACT) ^10^. Pandemic PACT aims to provide a more powerful, prospectively-designed and rolling research tool and analytical capacity for tracking research and evidence on epidemic-prone infectious diseases. The programme aims to support evidence-informed funding and research initiation to improve efficient use of limited research funds with the earliest possible information, contributing to strengthening the global health research system.

Here, we present the protocol for the grant funding tracking element of Pandemic PACT and our planned analyses through a LMR. We have built a FAIR-by-design ^11^ regularly updated original database of global funding for research on diseases with a pandemic potential, linked to publications through Europe PMC and aligned to relevant policy roadmaps. All related metadata and the database are made available in machine-actionable and user-friendly interface.

There are many two key questions which we aim to answer in this LMR using the Pandemic PACT data. Firstly, where are the gaps in the global distribution of research funding for infectious disease with a pandemic potential disaggregated by disease, geography, and research topic? Secondly, which funders are supporting infectious disease research (including clinical trials) globally and, specifically, in LMICs?

Further outbreak and policy roadmap specific questions will arise and these will be incorporated into our Pandemic PACT analyses outside of this protocol.

## Protocol

This protocol outlines the scope, content and methods used for the LMR with a specific focus on describing the design of the underlying global research funding database. We describe the search strategy and data extraction approaches used to collect the data and populate the database.

We intend to publish a baseline and update LMRs six-monthly for our Pandemic PACT grant funding database. The LMRs will provide regular analyses on the trends and alignment of the research funding landscape for infectious disease with a pandemic potential and broader pandemic preparedness. In the event of a future pandemic we will increase the frequency of our analyses for that particular disease to three-monthly (with fortnightly underpinning data collection) as was done for COVID-19 during the pandemic. Due to the prospective design, we anticipate minor changes to the upcoming LMR that have not been outlined in this protocol. Any changes will be detailed in the corresponding versions of the analysis, where appropriate.

This protocol complies to the PRISMA-P reporting guidelines. Checklist is available as Extended Data Checklist 1 ^12^.

### Rationale for using a living method

Research funding for infectious diseases is constantly evolving, with an anticipated continuing expansion of funding for ‘priority diseases’. Whilst some research funding is provided for basic research during the inter-epidemic periods, funding organisations also respond to global or regional outbreaks, by releasing new funding or repurposing existing grants. Moreover, in outbreaks, funding calls often have short time intervals, and funding allocation might be influenced by rapidly changing research needs and environment. Hence, to offer consistent near real-time data we will update the LMR regularly (every six months). As mentioned earlier, the case of a major outbreak, we will produce an update for that particular disease as a matter of priority within this already established system of living mapping reviews. In the event of a pandemic, we will shift to three-monthly updates of the LMR for that disease (as was undertaken during the COVID-19 pandemic).

### Eligibility criteria

Research grants funded by any non-commercial research funding organisation are eligible for inclusion, for the initial scope. We aim to include a full breadth of research themes with grants on pandemic preparedness and/or outbreaks focusing on; medical sciences and health, social sciences, ethics, surveillance, capacity strengthening and others.

***Start date.*** We will include grants with a start date on or after the 1 ^st^ of January 2020, to align with the research funding efforts relating to COVID-19, and hence the start date for the predecessor dataset from COVID CIRCLE ^5^. If no information on the award or start date was available for a grant identified, it will not be included in the initial version of the database. We may review this inclusion criteria further as the database develops to explore how to use other available information for those grants that are missing the award/ start date.

***Funders.*** For the initial version of the database, we are collecting available grant information from the funders of the GloPID-R and UKCDR memberships. The full list of these funders can be found in the Extended Data Table 1 ^12^. Further funders will be identified from the previous COVID-19 funding database, or their association with other funders, including a joint funding venture, or being a part of a network of funders, or other professional groups or relationships for inclusion in the baseline analysis.

***Diseases.*** We will initially include all diseases currently listed on the WHO R&D Blueprint priority disease list plus pandemic influenza, mpox and plague ^13^. These WHO R&D Blueprint priority diseases have been selected by WHO because they pose the greatest public health risk due to their epidemic potential or if there are no sufficient countermeasures to contain them. The list includes the following diseases: COVID-19; Crimean-Congo haemorrhagic fever; Ebola and Marburg virus disease; Lassa fever; Middle East respiratory syndrome coronavirus (MERS-CoV) and Severe Acute Respiratory Syndrome (SARS); Nipah and henipaviral disease; Rift Valley fever; Zika virus disease and Disease “X”. Disease “X”, is a concept rather than a specific disease, which represents “the knowledge that a serious international outbreak could be caused by a pathogen currently unknown to cause human disease”. Additionally, we will include three further important diseases (pandemic influenza, mpox and plague), on advice from our expert advisory group.

***Language.*** The search terms used are in English. However, we will not exclude grants in other languages. Hence, if our search returns any relevant grants in foreign languages, their title and abstract is translated using Google Translate, and they are included in the database. Other language search terms may be explored at a future date.

***Completeness of available grant information.*** We included all grant records containing a minimal level of essential information: grant award or start date or publication date; funder name; grant ID or other form of identifier or grant title.

### Data sources and search strategy

***Data sources.*** An inclusive and collaborative approach was applied to the data collection by holding consultations with the representatives from different funding organisations (open to members of the UKCDR and GloPID-R funder collaboratives) to agree on the preferred data collection modality. These consultations identified the need for flexibility in data collection, with some funders preferring the team to scrape data directly from their websites and others preferring to provide data directly. The data are therefore collected in one of the two ways, either by online search and automated or manual scraping from funder websites, which accounts for 64.7% of our funders (as of August 2024) and is 100% successful, or via direct data provision from some funders who either requested to directly contribute more comprehensive data than was currently available on their websites or do not have an online source for theirr data. This direct method accounts for 35.3% of our funders and has a success rate of about 33% (as of August 2024). Information about the source of the data is provided in the Extended Data Table 1 ^12^ for the initial database, but this will expand prior to the baseline analysis.

***Direct data submission.*** The database will remain open to new submissions related to the research grants for infectious diseases with pandemic potential from any non-commercial funder, via email and a custom-built data collection template (Extended Data Table 2 ^12^) and direct data upload route on Figshare). We will review all new submissions, include all relevant grants and update the database regularly.

***Search strategy.*** Search terms were developed and tested by working with colleagues in research funding organisations and other experts in the field (such as CIDRAP for Influenza). We included disease-specific keywords, acronyms, and expanded the terms to include the name of the virus and virus families ( Table 1).

**Table 1.** List of Diseases, Pathogens and Pathogen families included in the PANDEMIC PACT database and online tracker, including search terms used for data collection.

	Disease	SNOMED codes for diseases	Causative Pathogen	SNOMED codes for pathogens	Pathogen family	SNOMED codes for pathogen families	Search terms
1	Lassa virus infection	19065005	Lassa virus	85944001	Arenaviridae	243624009	Arenavirus, Lassa, LASV
2	Crimean-Congo haemorrhagic fever	43489008	Crimean-Congo Haemorrhagic fever virus	79875007	Bunyaviridae	243615000	Bunyavirus, Crimean-Congo, CCHF
3	Rift Valley Fever	402917003	Rift Valley fever virus	28335002	Bunyaviridae	243615000	Rift valley, RVF
4	Disease caused by severe acute respiratory syndrome coronavirus 2 (COVID-19)	840539006	Severe Acute Respiratory Syndrome Coronavirus 2	840533007	Coronaviridae	243607003	Coronavirus, Covid-19, SARS, SARS-Cov, MERS-Cov, nCoV, sarscov-2, Middle East respiratory syndrome, Severe Acute Respiratory Syndrome, coronavir
5	MERS-Middle East respiratory syndrome	651000146102	Middle East Respiratory Syndrome Coronavirus	697932005	Coronaviridae	243607003	
6	SARS-CoV infection/Severe Acute Respiratory Syndrome	398447004	Severe Acute Respiratory Syndrome Coronavirus (SARS-CoV1)	1263733001	Coronaviridae	243607003	
7	Ebola virus disease	37109004	Ebola virus	424206003	Filoviridae	407325004	Filovirus, Ebola, EBOV, Marburg, MARV, EVD, MVD, ebolavirus
8	Marburg virus disease	77503002	Marburg virus	424421007	Filoviridae	407325004	
9	Zika virus disease	3928002	Zika virus	50471002	Flaviviridae	243602009	Zika, ZIKV
10	Congenital infection caused by Zika virus	762725007	Zika virus	50471002	Flaviviridae	243602009	
11	Infection caused by Nipah virus	406597005	Nipah virus	115511007	Paramyxovirdiae	128355000	Henipavirus, Nipah, henipavir, hendra, NiVe
12	Hendra virus infection	773582002	Hendra virus	115510008	Paramyxovirdiae	128355000	
13	Other henipaviral disease	n/a	n/a	n/a	Paramyxovirdiae	128355000	
14	Influenza	6142004	Influenza A virus	407479009	Orthomyxoviridae	55014007	Orthomyxovirus, Influenza A subtype, pandemic influenza, H1N1, H5N1, H7N9, avian AND flu, zoonotic AND flu, swine AND flu
15	Mpox	359814004	Monkeypox virus	59774002	Poxviridae	424976006	Mpox, monkeypox, MPV, MPXV, hMPXV
16	Plague	58750007	Yersinia pestis	54365000	Yersiniaceae	1269429001	Bubonic Plague, Pneumonic Plague
17	Disease X	n/a	n/a	n/a	n/a	n/a	No specific key words to help with the search were identified

The search words were tested on a sub-set of funders’ research grant portals, namely the UK Research and Innovation ( https://gtr.ukri.org/), the National Institutes of Health ( https://reporter.nih.gov/), and Europe PMC ( https://europepmc.org/grantfinder/). A particularly challenging task was to identify grants for research on Disease “X”, because the search results returned grants for genetic conditions and non-communicable disease. To optimise the search results to overcome these challenges, we undertook manual screening of all research grants returned from the search to identify those that might be relevant. In addition to the disease-specific research grants, we were interested in covering a broad range of themes related to pandemic and outbreak preparedness, including infectious disease research capacity strengthening, surveillance and ethics, going beyond a named disease. The list of search terms is available in Table 1 and Table 2.

**Table 2.** List of search terms used for pandemic preparedness with a focus on capacity and surveillance.

Areas of interest	Search Terms
Capacity	Preparedness and novel pathogens, preparedness and pandemic, preparedness and novel infectious disease, clinical trials and pathogens of pandemic potential, clinical trials and pandemic, capacity and pandemic, capabilities and emerging infections ethics and pandemic ethics and novel infectious disease ethics and novel pathogens infrastructure and novel pathogens infrastructure and pandemic
Surveillance	Surveillance and novel pathogens, Data and novel pathogens, Data and pandemic, Disease surveillance and novel pathogens Modelling and novel infectious disease, Modelling and pandemic, Platforms and pandemic, Consortium and emerging infections

We are developing a Python code with these search terms, enabling us to query the backend of the grant databases and websites efficiently through API or by using web scraping technology like Selenium and WebDriver (code will be published on Git Hub once fully optimised).

### Data processes

**Figure 1.**
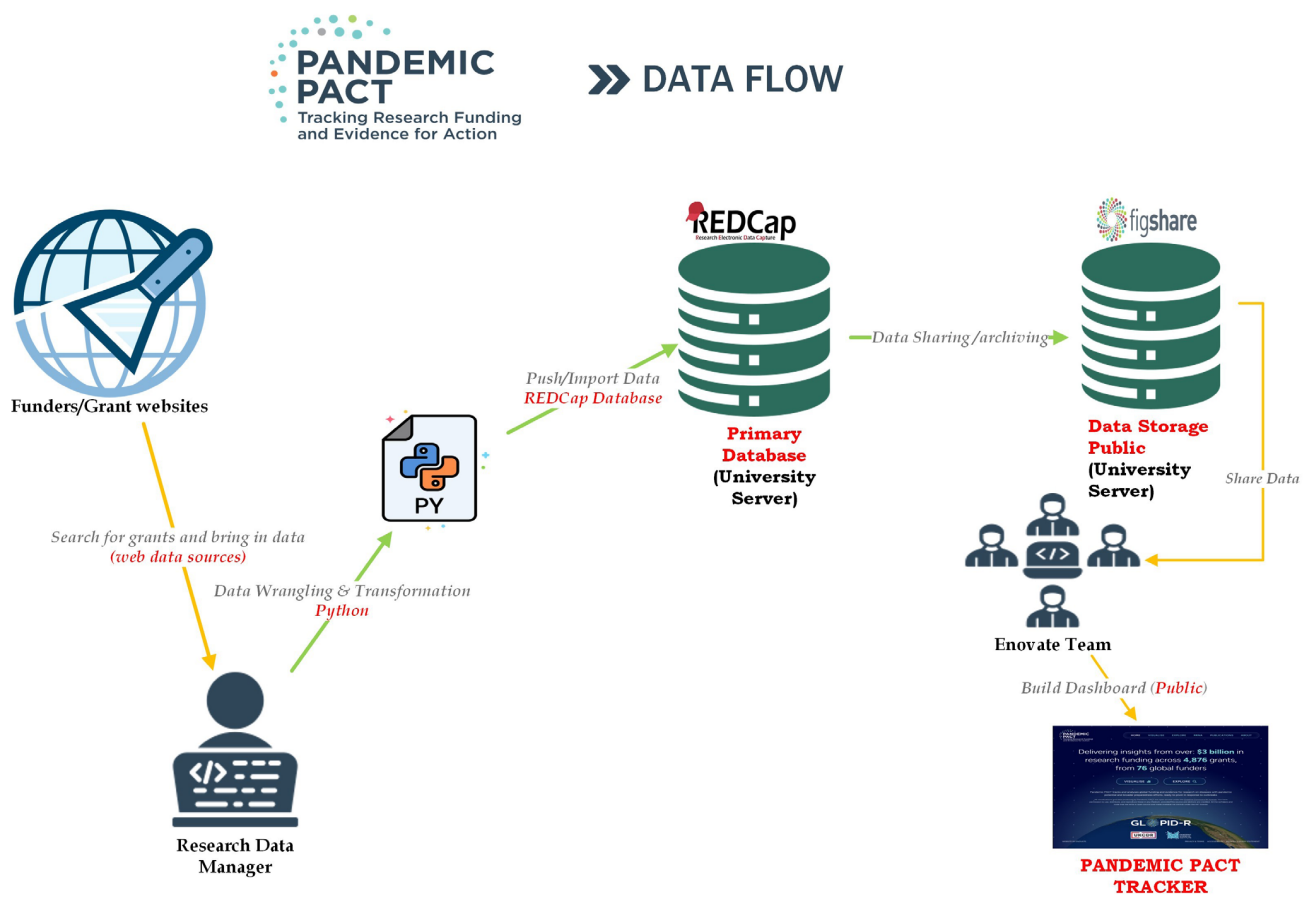
PANDEMIC PACT data flow and processes.

Figure 1 outlines our data flow and processes for the Pandemic PACT project. The data collection begins with searching for grants and bringing in data from various web data sources. Once collected, the data undergoes wrangling and transformation using Python. The transformed data is then pushed/imported into the REDCap database, which serves as our primary database.

To assess the relevance of the data, our team employs a combination of manual annotation, human coding, and machine-learning-assisted approaches. Initially, all collected data are reviewed by our researchers to determine their relevance. This involves examining the grant details, such as title, abstract, and funding information, to ensure they align with our inclusion criteria for infectious diseases within WHO priority diseases. Additionally, our team assigns appropriate research categories to each grant.

We utilise a Large Language Model (LLM) to augment the efforts of human annotators in classifying research projects into specified broad research categories. Specifically, we employ OpenAI model, which is capable of sophisticated natural language understanding. The methodology involves generating structured prompts based on the title and abstract of each project, which serve as concise summaries of the key project information. These prompts are then processed by the LLM using few-shot learning to accurately assign each project to primary and secondary research categories with minimal input examples

***Identifying and dealing with grant duplicates.*** To avoid creating multiple entries for the same grant in the database, we employ a series of de-duplication techniques. This is particularly important when acquiring data from different sources. Our goal is to ensure each grant is uniquely represented while also recording any grant supplements, which some funders may denote using the same Grant ID as the original grant.

Our de-duplication process has two main components

Automated Data Management:

○ We utilize automated scripts to cross-check new data against existing entries in the database.
○ The primary field used for detecting duplicates is the grant number assigned by the funder.
○ Additional fields such as the original grant amount, date of award, title, and abstract are also compared to identify potential duplicates.
○ These scripts flag records that have matching or closely similar details for further review.

Manual Revision by coding team:

○ A dedicated coder reviews the flagged records to confirm whether they are duplicates.
○ The coder examines the grant details, including any supplementary information, to ensure accurate identification.
○ This manual check helps in discerning true duplicates from records that may appear similar but are distinct.

By combining automated and manual approaches, we maintain the integrity of our database, ensuring it is both comprehensive and precise.

***Validation.*** Internal validation against the source data will be performed by our research team. We anticipate that the external validation of the database will be performed by individual funding organisations who will contrast and compare information captured and provided in the Pandemic PACT database against their own records. We aim to publish and report on any validation work undertaken in the future.

***Historical COVID-19 data and their transformation.*** The Pandemic PACT database expands upon the previous database co-developed by UKCDR and GloPID-R as part of the COVID-19 Research Coordination and Learning initiative (COVID CIRCLE). The database of funded COVID-19 research projects globally was launched in April 2020. By November 2023, the database contained 21.6k research projects worth (at least) $8.5 billion taking place across 161 countries ^5^. These projects were awarded by more than 370 funders around the world. Pandemic PACT represents the evolution of this work and has incorporated the majority of grants into its database (removing those which did not meet the eligibility criteria for Pandemic PACT as described above in order to ensure high-quality data), as well as expanded to include grants on the new chosen diseases beyond COVID-19. The COVID CIRCLE initiative has now ended and hence any data on recent COVID-19 grants will be made available in the Pandemic PACT database.

Among the unique characteristics of the COVID CIRCLE database was that all projects were mapped against the priorities identified in the WHO Coordinated Global Research Roadmap: 2019 Novel Coronavirus and the United Nations Research Roadmap for the COVID-19 Recovery. As Pandemic PACT covers diseases beyond COVID-19 work has been undertaken to update the classification framework, which was previously COVID-19 centred, to be more generalisable to a wider range of epidemic-prone infectious diseases. The new research categories are explained below.

To create a single, standardised database for the historical COVID-19 data and data on other diseases, we developed a new data schema, and used it to transform the historical COVID-19 grant data. Therefore, all old and new data are uniform and coded consistently to this framework.

***FAIR data.*** The database has been designed to align with the FAIR principles ^11^ to ensure our metadata and database are Findable, Accessible, Interoperable, and Reusable. In partnership with the GO FAIR foundation, we will publish a FAIR Implementation Profile (FIP) detailing our commitment to these principles, guiding our data stewardship and serving as a blueprint for others, especially those with limited resources.

A key aspect of our FAIR adherence is making all metadata and the database machine-actionable, enhancing data exploration. We've developed the Pandemic PACT FAIR Vocabulary, integral to our grant data schema, promoting a unified understanding and improving data usability across domains ^14^.

***Machine Learning.*** We have utilised a generative Large Language Model (LLM) to augment the efforts of human annotators in classifying research projects into specified broad research categories. The methodology involves generating structured prompts based on the title and abstract of each project, which serve as concise summary of the key project information. These prompts are then processed by the generative LLM, capable of sophisticated natural language understanding. By employing few-shot learning, the model is able to accurately assign each project to primary and secondary research categories with minimal input examples. This approach not only improves the accuracy of categorising a wide array of research data but also acts as a strategic complement to human annotation efforts, demonstrating our commitment to integrating cutting-edge techniques in the systematic evaluation of research projects.

### Database structure

***Data curation and management.*** The database is designed so that the unit of analysis is a research grant. The research grant is linked to the funding organisation, research organisation and a named investigator, if known. There can be multiple values for organisations and investigators for each grant.

Where possible, standardised lists and ontologies were used to populate variables to improve data interoperability ( Table 3). From the PubMed Central, SNOMED, ISO 3166-1 numeric and other standardised lists and vocabularies. In the database, we also recorded the names of funding organisations and research institutions using standardised lists – global list of funders (CrossRef ID) and Research Organisation Registry (ROR). We added an Open Researcher and Contributor ID (ORCID) (Ref https://orcid.org/) for the named investigators listed on the grant applications. In instances when no suitable standardised lists were identified, we adapted other popular ontologies and standardised nomenclature, including using PubMed MESH terms in the following variables – study subject, age group, rurality, vulnerable population, occupational group, clinical trial, ethnicity, country, region, research category, disease, pathogen, study type. Only a minority of variables were created empirically, based on our experience working with the COVID CIRCLE data. These are the broad research categories and research subcategories and tags.

**Table 3.** List of PANDEMIC PACT database variables and values with corresponding data format and data standards, and key notes.

N	Variable name	Data format	Data Standard	Values	Notes
1	PACTID	string	Non-standard, assigned internally	A combination of a letter character and numbers	
2	Grant in Scope	binary	Non-standard, assigned internally		
3	Grant Title Original	text	Non-standard		
4	Grant Title Eng	text	Non-standard		
5	Grant Number	text	Non-standard		As assigned by a funder
6	Grant Amount Original	string	Non-standard		
7	Grant Currency	string	ISO 4217 code		
8	Currency Exchange Rate USD	numeric	Non-standard		Calculated using API and code
9	Grant Amount Converted	numeric	Non-standard		
10	Grant Type	text	Non-standard		
11	Abstract Original	text	Non-standard		
12	Abstract English	text	Non-standard		
13	Lay Summary	text	Non-standard		
14	ODA funding used	binary	Non-standard, assigned internally		Official Development Assistance (ODA)
15	Grant Start Month	numeric	MM, ISO standard		
16	Grant Start Year	numeric	YYYY, ISO standard		
17	Grant End Month	numeric	MM, ISO standard		
18	Grant End Year	numeric	YYYY, ISO standard		
19	Publication Month of Award	numeric	MM, ISO standard		
20	Publication Year of Award	numeric	YYYY, ISO standard		
21	Grant Type	text	Non-standard	New Grant, Grant Extension	
22	Study Subject	Text, Boolean	MESH Terms	Animals, bacteria, human populations, disease vectors, viruses, environment, other, unspecified, not applicable	
23	Ethnicity	text, Boolean	Standard, UK Census	Asian, Black, White, Mixed, other, unspecified, not applicable	Optional field, populate if the grant is for research involving a specific ethnic group
24	Age Groups	Text, Boolean	MESH Terms modified	Adolescent, 13–17 yrs Adults, 18+ Children, 1–12 yrs, Infants, 1mth–1yr, Newborn (<1mth) Older adults, 65+ Unspecified, not applicable	Optional field, populate if the grant is for research involving a specific age group
25	Rurality	text, Boolean	MESH terms, modified	Rural population/setting, suburban population/setting, urban population/setting, other, unspecified, not applicable	Optional field, populate if the grant is for research on urban or rural populations or settings
26	Vulnerable Populations	Text, Boolean	MESH Terms, modified	Disabled persons, drug users, Internally Displaced and Migrants, Indigenous People, Sexual and gender minorities, Prisoners, Sex workers, Smokers, Women, Pregnant women, Individuals with multimorbidity, Minority communities unspecified, vulnerable populations unspecified, other, unspecified, not applicable	Optional field, populate if the grant is for research involving a specific vulnerable population group
27	Occupational Groups	Text, Boolean	MESH terms modified	Farmers, Emergency Responders, Military Personnel, Social workers, Caregivers, Health Personnel, Hospital personnel, Nurses and Nursing Staff, Physicians, Dentists and dental staff, Vets, Volunteers, other, unspecified, not applicable	Optional field, populate if the grant is for research involving a specific occupational group
28	Study Type	Text, Boolean	Non-standard	Clinical, Non-clinical, other, unspecified, not applicable	If clinical is selected, then there is an option to select a clinical trial phase and design and record this information in a new field. If non-clinical is selected, then there is an option to choose a report or literature review in a new field
29	Disease	numeric	Standard, SNOMED code	See the list of diseases. https://termbrowser.nhs.uk/	
30	Pathogen	numeric	Standard, SNOMED code	See the list of diseases. https://termbrowser.nhs.uk	
31	Funder	text	Standard, CrossRef Open Funder Registry	https://www.crossref.org/services/funder-registry/	
32	Funder Region	text	Standard, WHO region	https://en.wikipedia.org/wiki/List_of_WHO_regions	The region was assigned automatically based on the country of the funding organisation as listed in the global standard list
33	Funder Country	numeric	ISO 3166-1 numeric	https://www.crossref.org/services/funder-registry/	Country information was pulled from the CrossRef Open Funder Registry
34	Funder Acronym	text	Standard, CrossRef Open Funder Registry		Acronym was pulled from the CrossRef Open Funder Registry
35	Investigator Title	text	Non-standard		
36	Investigator First Name	text	Non-standard		
37	Investigator Last Name	text	Non-standard		
38	Investigator ORCID	string	Standard, ORCID ID number		Optional field. Researchers manually searched and entered the ORCID using the first and last name of the awardee.
39	ROR ID	string	Standard, ROR ID	https://ror.org/	Research Organisation Registry (ROR ID) for research institution
40	Institution Name	text	Standard, ROR list of research institutions	https://ror.org/	
41	Institution Country	text	Standard, ROR list of research institutions	https://ror.org/	
42	Institution Country ISO	numeric	ISO 3166-1 numeric	https://www.iso.org/iso-3166-country-codes.html	
43	Research Institution Region	text	Standard, WHO region		The region was assigned by a data manager using information from the ROR list
43	Partner Organisation Name	text	Non-standard		Information on the partner organisation is added if available in the grant abstract
45	Research Location Country	text	Non-standard		Information on the location of research is added if available in the grant abstract. Otherwise, we used the country where the Research Institution is based
46	Research Location Country ISO	numeric	Standard, ISO 3166-1 numeric code		
47	Research Location Region	text	Standard, WHO Region		Assigned based on the location of research is such information is available in the grant. Otherwise, we used the region where the Research Institution is based
48	Tags	Text, Boolean	Non-standard	Data Management and Data Sharing, Digital Health, Innovation, Gender	The tags were assigned by researcher who reviewed the grants
49	Research and Policy Roadmaps	Text, Boolean	Non-standard	100 Days Mission, WHO Surveillance, ESSENCE for Health	Mapping to selected roadmaps was done by researcher reviewing the grants
50	Primary Research Category	string	Non-standard	12 broad research categories, each has a list of subcategories	Researchers reviewed each grant and assigned a broad research category and subcategory. Multiple values permitted
51	Secondary Research Category	string	Non-standard	12 broad research categories, each has a list of subcategories	

Our researchers manually review all data entries to assign values to some variables in REDCap, when it was not possible to populate these fields automatically. The full list of variables and values are provided in the Extended Data Table 3 ^12^. We applied generic coding, unspecified or not available, across all variables when available information was no sufficient for interpretation of the data. No empty cells were permitted.

***Variables and Values.*** We provided a description for key variables here.

Grant Amount Original:

The total amount of research money is provided based on the amounts committed by the funders. Some funders do not publicly share the grant amounts, and we still include these records, but record this as information not available.

Grant Amount USD:

For our grant funding data processing, we convert all amounts to United States dollars using the `forex-python` library. This Python tool automates the retrieval of historical currency exchange rates directly from reputable sources like the European Central Bank, enabling us to convert grant amounts from their original currencies to USD accurately. By leveraging `forex-python`, we ensure our currency conversions reflect the exact value of each grant at the time it was awarded, providing a standardized and precise financial analysis across all grants.

Grant Start Year:

The field was populated with information on the year of when the grant was awarded where available. Otherwise, the field was coded as information not available.

Disease and Pathogen:

These two fields were populated using appropriate SNOMED codes for each infectious disease and the causal pathogen. The full list is available in Table 1. Unlike other diseases, Disease X was not assigned a SNOMED code, as none exists in the clinical classifications of diseases.

***Funder name and country.*** The name of the funding organisation and information about their country of affiliation was checked against the CrossRef Open Funder Registry, and then entered in the database. We recorded information on multiple funders if the grant was funded by a joint funding scheme.

***Funder Region.*** We assigned each funder into one of the six WHO regions based on the funder country. We added international and unspecified values for those that fell outside of these regions.

***Investigator first and last name.*** Where information about the grant awardee was available in the grant record, we entered the original first name and the last name into these two fields without any language translation (see data governance section below).

***Institution name and institution country.*** We checked the names of all institutions and organisations that were awarded funding against the global standardised list of Research Organisation Registry (ROR) ^15^, and entered the standardised name of the institution. Where no matches were found, for example, if the grant was given to non-research organisations, including private companies, the name was entered as provided in the grant. The country for research institution was assigned from the same global list. Then, the ISO code for the relevant country was recorded in the database.

***Institution region.*** We used the same approach for research institution, as for funding organisations. We assigned each research institution into one of the six WHO regions based on the institution country.

***Partner Organisation.*** We entered the name of the partner organisation if it was provided in the grant abstract in addition to the main research institution. The organisation name was entered exactly as provided in the grant. No curation was done on the organisation name. Multiple entries per individual grant were permitted.

***Research Location Country and Region.*** We entered information where the research activities are taking place if information about the country, or a specific location, was different from the location of the research institution, and was provided in the grant abstract. Multiple locations were permitted, and relevant ISO codes for countries were used. The countries were then grouped into the six WHO regions. In no additional information on the research location was available, we used that of the research institution.

***Research categories and subcategories.*** Building on lessons from tracking funding for COVID-19 research under the COVID CIRCLE initiative, we identified a lack of standard frameworks for classifying research on epidemic-prone diseases. Hence, we sought to identify areas for research classification which would remain stable over the breadth of diseases tracked under pandemic PACT.

We undertook a non-systematic review of broad health research categorisation systems including research roadmaps for other infectious diseases/ epidemic-prone diseases in the literature. This was complemented by a review of our approach to categorising COVID-19 research to identify emerging themes, which were not covered under the WHO COVID-19 Research Roadmap, which our work aligned to. We then undertook an iterative approach to refining and consulting on these categorisations including through a high-level workshop attended by a wide range of funders and policy stakeholders (including GloPID-R funder members, WHO and GAVI) in Annecy, France from the 31st January 2023 to 1st February 2023 and subsequent key stakeholder consultations (including with major funding bodies, and different teams within the WHO) (Extended Data Table 4 ^12^).

Where applicable we aligned definitions to WHO guidance on: community engagement; Health Policies and Systems Research; and, Vector control and ESSENCE guidance on capacity strengthening.

The resulting framework consists of 12 broad research categories with corresponding sub-categories, which are listed in Table 4 ^10^. While the broad categories show the overarching research themes, the subcategories provide further specificity/ details on research areas covered under each of the broad categories.

**Table 4.** Pandemic PACT research categories and subcategories.

Broad Research Categories	Research Sub-categories
1. Pathogen: natural history, transmission and diagnostics	a. Diagnostics
	b. Pathogen morphology, shedding & natural history
	c. Pathogen genomics, mutations and adaptations
	d. Immunity
	e. Disease models
	f. Environmental stability of pathogen
	g. N/A
	h. Unspecified
2. Animal and environmental research and research on diseases vectors	a. Animal source and routes of transmission
	b. Vector biology
	c. Vector control strategies
	d. N/A
	e. Unspecified
3. Epidemiological studies	a. Disease transmission dynamics
	b. Disease susceptibility
	c. Impact/effectiveness of control measures
	d. Disease surveillance & mapping
	e. N/A
	f. Unspecified
4. Clinical characterisation and management	a. Prognostic factors for disease severity
	b. Disease pathogenesis
	c. Supportive care, processes of care and management
	d. Post acute and long-term health consequences
	e. Clinical trials for disease management
	f. N/A
	g. Unspecified
5. Infection prevention and control	a. Restriction measures to prevent secondary transmission in communities
	b. Barriers, PPE, environmental, animal and vector control measures
	c. IPC in health care settings
	d. IPC at the human-animal interface
	e. N/A
	f. Unspecified
6. Therapeutics research, development and implementation	a. Pre-clinical studies
	b. Phase 0 clinical trial
	c. Phase 1 clinical trial
	d. Phase 2 clinical trial
	e. Phase 3 clinical trial
	f. Phase 4 clinical trial
	g. Prophylactic use of treatments & Repurposed drugs
	h. Clinical trial (unspecified trial phase)
	i. Therapeutics logistics and supply chains and distribution strategies
	j. Therapeutic trial design
	k. Adverse events associated with therapeutic administration
	l. N/A
	m. Unspecified
7. Vaccines research, development and implementation	a. Pre-clinical studies
	b. Phase 0 clinical trial
	c. Phase 1 clinical trial
	d. Phase 2 clinical trial
	e. Phase 3 clinical trial
	f. Phase 4 clinical trial
	g. Clinical trial (unspecified trial phase)
	h. Vaccine logistics and supply chains and distribution strategies
	i. Vaccine design and administration
	j. Vaccine trial design and infrastructure
	k. Adverse events associated with immunization
	l. Characterisation of vaccine-induced immunity
	m. N/A
	n. Unspecified
8. Research to inform ethical issues	a. Research to inform ethical issues in Research
	b. Research to inform ethical issues related to Public Health Measures
	c. Research to inform ethical issues in Clinical and Health System Decision-Making
	d. Research to inform ethical issues in the Allocation of Resources
	e. Research to inform ethical issues in Governance
	f. Research to inform ethical issues related to Social Determinants of Health, Trust, and Inequities
	g. N/A
	h. Unspecified
9. Policies for public health, disease control & community resilience	a. Approaches to public health interventions
	b. Community engagement
	c. Communication
	d. Vaccine/Therapeutic/ treatment hesitancy
	e. Policy research and interventions
	f. N/A
	g. Unspecified
10. Secondary impacts of disease, response & control measures	a. Indirect health impacts
	b. Social impacts
	c. Economic impacts
	d. Other secondary impacts
	e. N/A
	f. Unspecified
11. Health Systems Research	a. Health service delivery
	b. Health financing
	c. Medicines, vaccines & other technologies
	d. Health information systems
	e. Health leadership and governance
	f. Health workforce
	g. N/A
	h. Unspecified
12. Research on Capacity Strengthening	a. Individual level capacity strengthening
	b. Institutional level capacity strengthening
	c. Systemic/environmental components of capacity strengthening
	d. Cross-cutting
	e. N/A
	f. Unspecified

Each grant was assigned to one or multiple main broad categories and subcategories. Subsequently, grants with additional subordinate research aim were allocated to one or more secondary categories with corresponding subcategories. The assignment was based on the information provided in the grant's title and abstract. Where there was insufficient grant information for classification of the main broad category, projects were assigned to 'unspecified'. If grants did not align with any of the broad categories, they were assigned to ‘not applicable (N/A)’.

For the sub-categories, the assignment of ‘unspecified’ was made if grants met the inclusion criteria of the broad category areas(s) but lacked sufficient information for further classification of subcategories. When grants clearly fell outside the established subcategories they were assign to 'N/A'.

To facilitate the coding process, a coding guide was developed (Extended Data Table 3 ^10^). We refine the descriptions for the subcategories through an iterative process and weekly discussions involving researchers engaged in coding.

***Cross-cutting tags.*** To identify grants that cover areas of particular interest for funding organisations and policy makers, we introduced a number of cross-cutting tags to which the relevant projects were assigned. These are Digital Health; Innovation; Gender; and, Data Management and Data Sharing.

***Research and Policy roadmaps.*** We are committed to aligning research grants to relevant existing and upcoming research agenda and policy frameworks. This will demonstrate the level of alignment between the global research funding and the policy work. Our researchers will manually assign grants to relevant roadmaps if they were within the scope, as a binary variable and we will then develop more specific coding frameworks within the roadmaps. The current frameworks planned for inclusion are the 100 Days Mission, WHO Surveillance and ESSENCE for Health. We are looking to expand the number of frameworks considered in the future. The analyses of these alignments will however fall beyond the scope of this LMR protocol. 

### Data analysis plan

We are planning to publish a baseline LMR with subsequent six-monthly updates. The data analysis will be conducted using software with analytical capabilities such as STATA, R, and Microsoft Excel. We will publish descriptive statistics, figures, and summary tables, as well as discuss our findings in the global context of research funding and policy work. Specifically, we anticipate publishing analyses examining the trends in financial commitments to research on epidemic-prone infectious diseases. These analyses will detail the variability in grant amounts, including the range from minimum to maximum grant awards and the total known commitment for each disease. Importantly, to identify gaps in the global distribution of research funding, the LMR will regularly update the allocation of funds by research priority areas. In doing so, we will provide an overview of the current landscape, highlighting not only the total known financial commitments for each disease but also the specific types of research being funded and areas where funding is lacking.

Additionally, our baseline LMR will provide a more fine-grained analysis of the characteristics of the funding, such as the geographical location of funding organisations and the research institutions receiving the awards. By tracking the geographical flow of commitments, we will shed light on existing inequalities in the distribution of funds, particularly focusing on the proportion of investments directed towards LMICs. Our analyses will not only summarise the total commitments flowing within and between regions but also illustrate how these patterns evolve over time.

Our regularly updated analyses will also delve deeper into the characteristics of the awarded projects. This includes the type of research being conducted and the study populations involved. We will offer a summary of the total known financial commitments to clinical research, detailing the investment committed to clinical trials and identifying its distribution across different trial phases. Moreover, by leveraging the richness of Pandemic PACT data, we will identify the study populations (e.g., humans, animals, viruses) involved in the research funded. Human populations will be further scrutinised in terms of age groups and vulnerability status, among other sociodemographic factors, to identify which specific population groups have been involved in funded research.

### Data governance

For the purposes of the Pandemic PACT project, we have collected data on researchers and their research outputs using names and Open Researcher and Contributor IDs (ORCIDs) (“personal” data). The University of Oxford are the ‘data controller’ for these data, which means we decide how to use it and are responsible for looking after it in accordance with the UK General Data Protection Regulation and associated data protection legislation. We share data with anyone who wishes to download and re-use the information under a CC-BY licence. We will only retain data for as long as we need it to meet our purposes, including any relating to legal, accounting, or reporting requirements. Data will be held securely in accordance with the University’s policies and procedures. Further information is available on the University’s Information Security website where information on rights in relation to personal data are explained.

### Data updates and archiving

The public database, all visualisations informed by these data, and the living mapping review, will be updated at least every six months. At pre-arranged dates, our team will release the updates to the database, with the respective changes to all online resources, as near-real time snapshots of the funding activities recorded up to that point within a calendar year. The frequency of updates will be consistent, with only new grants added to the existing database to avoid duplication. This work will continue for as long as the PANDEMIC PACT programme is funded. Historical copies of the database will be marked accordingly and remain available on the Figshare platform.

## Discussion

Among the key lessons reinforced by the COVID-19 pandemic is the ongoing need for good-quality data. Just as epidemiological and virological data provide vital information on the severity and extent of disease spread, funding data, along with research evidence data, can inform decisions regarding research investment allocation. For its part, Pandemic PACT seeks to contribute to this knowledge gap by providing reliable funding data for research on epidemic-prone infectious diseases and broader research preparedness efforts. The protocol presented here represents a new approach to rapidly collating, coding, and analysing grant funding data for pandemic preparedness. Our subsequent living analyses of these data to be presented in this living review will address our key research questions.

By providing a systematic review of research investments, Pandemic PACT signifies the value of funding data as one key component in a broader ecosystem of understanding the pandemic preparedness and response in retrospect and considering how decision-making can be improved for better outcomes in a future global health crisis. In this context, Pandemic PACT’s key strength lies in addressing the growing demand for this type of data and analysis, acknowledging the importance of publicly accessible funding information that is, consistent easily digestible and provided in as close to real time as possible. This protocol itself aims to enhance transparency, demonstrate data quality methodologies, and facilitate easy access to comprehensive project documentation. Such efforts are crucial for fostering collaboration and coordination, particularly during non-pandemic periods.

We will continue to update and innovate this approach over time to ensure the best possible use of these data for evidence-informed decision making. These grant funding data will also be combined with other data sources to enrich our future analytical work. Whilst we will launch the living database for this work in March 2024, we intend to publish the baseline LMR analysis in Summer 2024.

### Limitations

***Database.*** Among the main limitations of the database is the varying completeness of data which can lead to less refined categorisation (assignment of projects to broad priority but not sub-priority areas) where the qualitative details of projects provided were insufficient. Therefore, assigned priority areas may have failed to capture all aspects of the projects relevant to existing and upcoming policy frameworks. The same can be said for any value that was assigned to a given research project by the project team, including all aspects related to the study population and study type. The data validation process by reviewers with expertise in global health research, policy, and funding is used to address this and ensure that any assigned value was as accurate as possible, given the information provided.

Data on funding amounts is not available from all funders and as a result this database is limited in providing a full financial profile of priority diseases funding investments. However, as the database will make use of all publicly available information, it can therefore be considered the most comprehensive possible.

At a higher level, the comprehensiveness of the database is limited to the funders that have either provided data for the database or had their data extracted from online sources (if available) and by the quality of that available data. In this respect, there were challenges in engaging with (and obtaining data from) health research funders beyond existing networks either due to a lack of contacts or capacity from funders to contribute to the project (especially for funders whose award information is not in English). This challenge is exacerbated by the dynamic nature of the database, which is continuously expanding to accommodate the ever-evolving landscape of research funding for infectious diseases with a pandemic potential.

***Risk of Bias.*** This protocol of funded research projects on infectious diseases with a pandemic potential uses descriptive and thematic analysis to summarise the scope of funded research projects. No attempts are made to assess the quality of individual studies or whether the studies meet their objectives. The potential sources of bias with project selection, quality of data reviewed, and data extraction and classification are addressed by robust fortnightly searches, template completion by funders and independent assessment and review during project classification respectively, as mentioned in the Information Sources and Search Strategy.

While the intention of the database and subsequent analyses are to provide as comprehensive a picture as possible of the landscape of research on diseases with a pandemic potential, the data obtained for the database is more likely to be derived from funders of research that are members of UKCDR (all UK and broad disciplinary focus) and/or GloPID-R (global membership spanning high-income countries, or HICs, to low-income countries, or LICs, with a majority of national funders, and a biomedical focus). This will likely skew the results to show that more research being funded from these organisations and reflect trends in their respective portfolios (in terms of location, research focus and research activity type) than may necessarily be the case of the landscape more generally.

An important limitation of the protocol is its inability to anticipate future challenges, particularly in light of the dynamic nature inherent to infectious disease outbreaks. Therefore, this protocol acknowledges that unforeseen challenges may emerge, necessitating adjustments, incorporations, or developments of more efficient data collection methods and coding strategies. To address this limitation, we commit to maintaining an adaptive approach, consistently updating the documentation of the database to accommodate modifications, incorporations, or discontinuations in response to evolving circumstances. Transparency will be maintained by accessible public documentation outlining all alterations and integrated processes.

## Ethics and consent

Ethical approval and consent were not required.

## Data Availability

The continuingly updated data related to this study are openly available on Figshare platform at: https://figshare.com/s/9e94a0e039df18316faa. All code will be made available on GitHub. The data can also be explored and exported on the online public dashboard, which includes high quality figures and charts, also available for downloading. Figshare: Extended data for: 'A protocol for a living mapping review of global research funding for infectious diseases with a pandemic potential – PANDEMIC PACT'. https://doi.org/10.25446/oxford.c.7112065 ^12^. This project contains the following extended data: Table 1. List of Diseases, Pathogens and Pathogen families included in the PANDEMIC PACT database and online tracker, including search terms used for data collection. Table 2. List of search terms used for pandemic preparedness with a focus on capacity and surveillance Figure 1. Pandemic PACT data flow and processes Table 3. List of PANDEMIC PACT database variables and values with corresponding data format and data standards, and key notes. Table 4. Pandemic PACT research categories and subcategories Extended Data Table 1. List of GloPID-R and UKCDR member funders and sources of data. Extended Data Table 2. PANDEMIC PACT Data Collection Template for Direct Data Provision Extended Data Table 3. PANDEMIC PACT Data Coding Guidance Extended Data Table 4. List of Organisations participating in the PANDEMIC PACT Tool and Research Categories Development meeting in Annecy, France, February 2022. Extended Data Checklist 1. PANDEMIC PACT PRISMA-P reporting guidelines Data are available under the terms of the Creative Commons Attribution 4.0 International license (CC-BY 4.0).
