# Supplementary material for: A protocol for a living mapping review of global research funding for infectious diseases with a pandemic potential – Pandemic PACT
Source: Wellcome Open Res. 2024 Oct 2;9:156. Originally published 2024 Mar 19. [Version 2] doi: 10.12688/wellcomeopenres.21202.2 (PMC11487232; doi:10.12688/wellcomeopenres.21202.2)
Supplement: Peer review history

# Reviewer report: Reviewer response for version 2

**DOI:** 10.21956/wellcomeopenres.25384.r103023

**Published:** 2024-10-22

**Authors:** Bernardo Aguilera

**Affiliations:** 1 Universidad San Sebastian, Santiago, Chile

The authors present a protocol for a grant funding tracking system for grants related to infectious diseases with epidemic/pandemic potential. This would be an important contribution. A comprehensive and updated database about what research is being funded globally is crucial to avoid waste and duplication. It would also be an important step in ensuring a fair global distribution of research resources. Overall, I think this is a valuable project, however some passages are not easy to read and the writing could be improved (in particular, the discussion section). Below, I offer some more specific comments.

 The acronym living mapping review (LMR) is introduced in the abstract but not in the main text, as it should be before it is being used. Beyond that, it is worth noting that dubbing the present database a "living mapping review" could be an overstatement, given that it will be updated every six months. For a "living" database I would expect much more regular updates.

 In "Eligibility criteria" the authors state that they will only include research grants funded by non-commercial research funding organizations. I wonder if they should mention this as a limitation, given that most clinical research worldwide is funded by commercial sponsors.

 To create the database the authors will use search terms in English. I wonder if this means that databases in languages with non-Latin alphabets would then be excluded. If so, then this should be considered a limitation.

 In "Research categories and subcategories" the authors say that if grants did not align with any of the broad research categories, they were assigned to ‘not applicable (N/A)’. This seems problematic since without categorizing the grant under any label the authors would not be in a position to determine if it should be included in their database, since it is supposed to track only "grants on pandemic preparedness and/or outbreaks".

 In the same section the authors state that "We refine the descriptions for the subcategories through an iterative process and weekly discussions involving researchers engaged in coding." This is written in present tense. Does it mean that the process of refining the descriptions of the categories is ongoing? (Table 4, on the contrary, suggests that the categories have been settled).

 In "Research Policy roadmaps", the authors claim that their goal (one that goes beyond the present protocol, however) is to align "research grants to relevant existing and upcoming research agenda and policy frameworks" and to "regularly update the allocation of funds by research priority areas". How will those priority areas be determined? Would they correspond to those established by international non-commercial entities, such as WHO? Would they include private entities? What about priorities that have been set at national/regional levels? More clarity about this part of the project would help visualize the project's potential.

 In the third paragraph of "Data analysis plan" the authors write: "we will identify the study populations (e.g., humans, animals, viruses) involved in the research funded". If they mean research participants, I think they should only be including human and non-human animals. It seems odd to consider "viruses" (or cells, or tissues, for that matter) as study populations.

 In "Limitations", first paragraph, the authors say: "The same can be said for any value that was assigned to a given research project by the project team, including all aspects related to the study population and study type." As I understand, the authors are using "value" in a computer science sense to refer to the numerical magnitude assigned to a given research project. I would suggest, however, to use a different term, since "value" in the present context is likely to be understood as the worth or importance that is assigned to the research project (i.e. normative sense).

 In the same section, where they say: "The potential sources of bias with project selection, quality of data reviewed, and data extraction and classification are addressed...", I think "quality of data reviewed" sounds more like a result of biases rather than a source for them.

Is the study design appropriate for the research question?

Yes

Is the rationale for, and objectives of, the study clearly described?

Partly

Are sufficient details of the methods provided to allow replication by others?

Yes

Are the datasets clearly presented in a useable and accessible format?

Not applicable

Reviewer Expertise:

Normative and empirical bioethics

I confirm that I have read this submission and believe that I have an appropriate level of expertise to confirm that it is of an acceptable scientific standard, however I have significant reservations, as outlined above.

# Reviewer report: Reviewer response for version 2

**DOI:** 10.21956/wellcomeopenres.25384.r102664

**Published:** 2024-10-17

**Authors:** Sam Halabi

**Affiliations:** 1 Georgetown University, Washington, USA

My comments have been addressed. It is indexable from my perspective.

Is the study design appropriate for the research question?

Yes

Is the rationale for, and objectives of, the study clearly described?

Yes

Are sufficient details of the methods provided to allow replication by others?

Partly

Are the datasets clearly presented in a useable and accessible format?

Partly

Reviewer Expertise:

Scoping review methods in public health law

I confirm that I have read this submission and believe that I have an appropriate level of expertise to confirm that it is of an acceptable scientific standard.

# Reviewer report: Reviewer response for version 2

**DOI:** 10.21956/wellcomeopenres.25384.r102663

**Published:** 2024-10-17

**Authors:** Mario Coccia

**Affiliations:** 1 Arizona State University, Tempe, Arizona, USA

After the revision, I believe the contribution has improved.

Is the study design appropriate for the research question?

Partly

Is the rationale for, and objectives of, the study clearly described?

Partly

Are sufficient details of the methods provided to allow replication by others?

Partly

Are the datasets clearly presented in a useable and accessible format?

Partly

Reviewer Expertise:

COVID-19,  Crisis management

I confirm that I have read this submission and believe that I have an appropriate level of expertise to confirm that it is of an acceptable scientific standard.

# Reviewer report: Reviewer response for version 2

**DOI:** 10.21956/wellcomeopenres.25384.r103024

**Published:** 2024-10-07

**Authors:** Richard Kock

**Affiliations:** 1 RETIRED, The Royal Veterinary College Department of Pathobiology and Population Sciences Hawkshead Campus, London, England, UK

The authors are proposing a future proofed development of a review method for live research funding for infectious diseases with pandemic potential. This was based on efforts during the COVID outbreak where significant funding and collective research effort created a large, dynamic and somewhat chaotic research field and output. he basis for this is around WHO approved diseases frequently described to have pandemic potential as well as agent X an unknown pathogen expected. They make every effort to create a method for detection of funders globally and means to access data and information from these funders against the disease criteria they have set. The system is flexible in order to address accelerated demands associated with a pandemic. The paper has already been adjusted after initial 2 reviews which had reservations.

 The rationale for developing the new method  is explained and no basic fault can be found as it is fairly obvious that in the event of future pandemics, resources need to be well targeted to prevent such an event, and in the case of such an event the preparedness needs sound foundations globally as well as a competent response capacity and approach. The description of the method is technically sound and considerable effort has been made in the paper to explain every nuance of the approach. Again no fault in this. There would be no problem in retesting the method to assess its validity for the purpose described by another group. The only weakness that I see in this paper, relates to its comprehensiveness in the context of the objective of improved preparedness and response to pandemics.  The conclusions about the method and its performance are not adequately supported by any findings presented in the article other than for COVID? For the other diseases specified there is little or no information to justify that this approach will be beneficial. There is also a gap in my opinion around some of the Key Research areas associated with the chosen diseases. They are very conventional and medical in their approach to infectious disease with some attention to general public health aspects and other social implications or consequences but there is virtually no attention given to the underlying drivers and risk factors for these diseases. The latter is probably much more important than the preparedness and response components. For example for Nipah and Henipah viruses, we now understand the ecologies and epidemiologies of these emergent pathogens and research in the area of mitigating the emergence are not mentioned. Resolution of this potential problem does not lie in preparedness or response but in ensuring ecological stabilities through better land management and human domain development within given environments and host pathogen communities. This is also true for other of the key diseases mentioned. I feel this gap is so important in terms of the research funding domain that without its inclusion the approach will be of some but less than useful impact. I would ask the authors to review this aspect and if they are able to include a broader set of research funding criteria and parameters to search including epidemiological, ecological, environmental, socioeconomic and development research data and funding in the context of disease, it will close the gap and make this extremely useful an approach indeed.

Is the study design appropriate for the research question?

Partly

Is the rationale for, and objectives of, the study clearly described?

Partly

Are sufficient details of the methods provided to allow replication by others?

Yes

Are the datasets clearly presented in a useable and accessible format?

Yes

Reviewer Expertise:

Infectious disease epidemiology in situ. One Health approach with a focus on wildlife diseases and at the wildlife livestock human interface. Understanding disease emergence in novel hosts, research challenges and consequences. Veterinary focus.

I confirm that I have read this submission and believe that I have an appropriate level of expertise to confirm that it is of an acceptable scientific standard, however I have significant reservations, as outlined above.

# Reviewer report: Reviewer response for version 1

**DOI:** 10.21956/wellcomeopenres.23449.r83691

**Published:** 2024-06-04

**Authors:** Sam Halabi

**Affiliations:** 1 Georgetown University, Washington, USA

The rationale and objectives of the protocol are both clear and justified. There was during COVID, and continues today, duplication and poor matching between researchers and funders. Emergencies exacerbate this problem. The researchers pose the following questions, not all of which appear directly answerable by the protocol assembled:

 Where are the gaps in the global distribution of research funding for infectious disease with a pandemic potential? To what extent is research funding aligned to the major research agendas and policy frameworks? Which funders are supporting infectious disease clinical trials globally and, specifically, in LMICs? 

 The study (PANDEMIC PACT) is also appropriate for the research question, focused as it is on funders of the diseases designated. However, the protocol’s description and methods could be made more practicable with additional details on some aspects.

 Regarding text around Table 1, I tried to navigate to footnote 10’s list of full funders and was unable to locate it.

 With respect to *Data Sources*, the following sentence is representative of some of the detail that is currently lacking but could be provided in the interest of duplicability and transparency: “An inclusive and collaborative approach was applied to the data collection by holding consultations with the representatives from different funding organisations to agree on the preferred data collection modality.” Without specification of how inclusion and collaboration (and with whom) was achieved, it is difficult for the reader or user to scrutinize the applied modality.

 This is similar for “direct data provision from a minority of funders.” How was this direct data solicited? What constitutes a “minority”? For the latter, this number could be provided in discrete terms.

 With respect to the text around Figure 1, the protocol would be improved by specifying how the team assesses relevance and which LLM is applied.

Is the study design appropriate for the research question?

Yes

Is the rationale for, and objectives of, the study clearly described?

Yes

Are sufficient details of the methods provided to allow replication by others?

Partly

Are the datasets clearly presented in a useable and accessible format?

Partly

Reviewer Expertise:

Scoping review methods in public health law

I confirm that I have read this submission and believe that I have an appropriate level of expertise to confirm that it is of an acceptable scientific standard, however I have significant reservations, as outlined above.

# Response

**Published:** 2024-08-29

**Authors:** Alice Norton

**Affiliations:** University of Oxford, UK

Many thanks for this helpful and constructive review.

 We have now submitted an updated version taking on board comments from yourself and Reviewer 1. We will also shortly be submitting our baseline review of the dataset. 

 We have clarified the protocol further, to demonstrate more clearly how the questions posed will be answered by the protocol. 

 We have added further details in the methods to make them more specific (to the extent possible for a protocol) as recommended.

 We have also addressed the issues with the text and content around Table 1 and have improved the text relating to Figure 1.

# Reviewer report: Reviewer response for version 1

**DOI:** 10.21956/wellcomeopenres.23449.r79074

**Published:** 2024-05-28

**Authors:** Mario Coccia

**Affiliations:** 1 Arizona State University, Tempe, Arizona, USA

A protocol for a living mapping review of global research funding for infectious diseases with a pandemic potential –PANDEMIC PACT

 The topics of this paper are interesting. The content must be revised, and results have to be better explained by authors.

 Title has to be shorter. 

 Abstract has to describe the method about how this protocol can support an effective health policy, associated with other factors, to face any future pandemics.

 Introduction has to better clarify the research questions of this study and provide more theoretical background about COVID-19, and strategies to cope with  pandemics, considering the differences between HICs and LMICs. After that authors can focus on the topics of this study to provide a correct analysis for fruitful discussion. (See suggested readings that must be all read and used in the text). 

 In the protocols section, I suggest the authors to divide the period into two: with and without pandemic, because even updating the LMR regularly (Every 6 months), in the pandemic crisis, will not be of much use in face of rapid emergence of variant mutant of viral agents. Real-time have to be monthly in pandemic crisis.

 PRISMA approach can be represented with a graph to be clearer for readers. 

 Tables 1 and 3-4 are long and difficult to digest in text, creating dispersion. I suggest putting them in the appendix. 

 Discussion. 

 First, authors have to synthesize the main results in a simple table to be clear for readers and then show what this protocol adds compared to other studies. I suggest inserting a SWOT matrix to show pros and cons of the PROTOCOL, as well as to divide the PANDEMIC PACT tools in a context of strategy with and without pandemic crisis in society, when time and events occur with different frequencies and intensity in a short period of time.  

 Authors have to show how this approach is inserted in an ecosystem associated with other elements and institutions that have to be prepared to face pandemic crisis with appropriate strategic implementation. 

 Now it is not clear. 

 One of the problems is the data updates that have to be monthly to be really timely for rapid decision-making processes in pandemic crisis when in a short period occurs different events.

 PANDEMIC PACT seems to have a potential utility but practical utility has to be proved in contexts of crisis management when a lot of social and institutional factors play a vital role in turbulent environments.

 I suggest inserting PANDEMIC PACT in a clear ecosystem based on Repository, technologies to manage data, skilled human resources, and good governance in institutions, clarifying who does what and what do in context of crisis management. 

 Conclusion has not to be a summary, but authors have to focus on manifold limitations of this study and how to test the utility in practical context of crisis management.  

 UK and USA were considered in a rank at the top to cope with pandemic crisis, before COVID-19, but facts have shown different weaknesses for manifold factors on-going during a pandemic.

 So short updated of data (monthly) and timely interventions are main aspects to consider. 

 Overall, then, the paper is interesting. Theoretical framework is weak, and some results create confusion for practical implementation of the protocol … study design, discussion and presentation of results have to be clarified using suggested comments.

 I strongly suggest improving the paper,  by using all comments (suggested papers included to read and use all) that I will in-depth verify. 

 Suggested readings of relevant papers that can be read and used.

 Coccia M. 2021. (Ref 1)

 Kwon, S. et.al., 2024 (Ref 2)

 Coccia M. 2023. (Ref 3)

 Bayly, H., et.al 2024 (Ref 4)     

 Benati I.,et.al., 2022 (Ref 5)

 Proposed articles can support the theoretical background of proposed protocol for improving the discussion and insert the protocol in a systematic context, associated with other actions, interventions and tools,  of pandemics prevention and management in the presence of future new viral agents and related variants.

Is the study design appropriate for the research question?

Partly

Is the rationale for, and objectives of, the study clearly described?

Partly

Are sufficient details of the methods provided to allow replication by others?

Partly

Are the datasets clearly presented in a useable and accessible format?

Partly

Reviewer Expertise:

COVID-19,  Crisis management

I confirm that I have read this submission and believe that I have an appropriate level of expertise to confirm that it is of an acceptable scientific standard, however I have significant reservations, as outlined above.

## References

[1] : Pandemic Prevention: Lessons from COVID-19. *Encyclopedia* .2021;1(2) : 10.3390/encyclopedia1020036 433-444 10.3390/encyclopedia1020036

[2] : General protocol for predicting outbreaks of infectious diseases in social networks. *Sci Rep* .2024;14(1) : 10.1038/s41598-024-56340-7 5973 10.1038/s41598-024-56340-7 38472283 PMC10933380

[3] : Sources, diffusion and prediction in COVID-19 pandemic: lessons learned to face next health emergency. *AIMS Public Health* .2023;10(1) : 10.3934/publichealth.2023012 145-168 10.3934/publichealth.2023012 37063362 PMC10091135

[4] : Looking under the lamp-post: quantifying the performance of contact tracing in the United States during the SARS-CoV-2 pandemic. *BMC Public Health* .2024;24(1) : 10.1186/s12889-024-18012-z 595 10.1186/s12889-024-18012-z 38395830 PMC10893709

[5] : Global analysis of timely COVID-19 vaccinations: improving governance to reinforce response policies for pandemic crises. *International Journal of Health Governance* .2022;27(3) : 10.1108/IJHG-07-2021-0072 240-253 10.1108/IJHG-07-2021-0072

# Response

**Published:** 2024-08-29

**Authors:** Alice Norton

**Affiliations:** University of Oxford, UK

Many thanks for this review. We have now submitted an updated version taking on board comments from both yourself and Reviewer 2. We will also shortly be submitting our baseline review of the dataset. 

 In specific response to your comments: We have improved the clarity of the abstract and introduction, and ensured that the research questions are more clearly defined. Each of the articles from the provided list of suggested papers have been reviewed and, through making some engaging wider points, it was decided that they were not directly relevant to cite in this publication. We have however made changes to improve the positioning of this work amongst the relevant literature and included further relevant citations. 

 We have now strengthened the multiple references in the protocol to the planned methodological adaptions to the protocol in the event of a substantial outbreak or pandemic. The PRISMA diagram format already used in the manuscript is based on existing best practice (Joanna Briggs Institute). 

 We have decided to leave the tables within the main text due to their central relevance to the protocol. Both the online and PDF versions of the article allow easy interpretation of these tables alongside the relevant corresponding text. Given this is a protocol paper there are no results to synthesise in the discussion. We have however taken on board the suggestion to expand the discussion and also to show greater delineatrion between pandemic and non-pandemic times in this section. We have also provided further positioning for this work in the ecosystem landscape, as suggested.
